# WAR COHORT DIFFERENCES IN MILITARY SERVICE APPRAISALS AND HOMECOMING EXPERIENCES

**DOI:** 10.1093/geroni/igz038.2566

**Published:** 2019-11-08

**Authors:** Dylan Lee, Soyoung Choun, Maria Kurth, Hyunyup Lee, Carolyn M Aldwin

**Affiliations:** 1 OSU Oregon State University, Corvallis, Oregon, United States; 2 Oregon State University, Corvallis, Oregon, United States

## Abstract

Nearly all the research on appraisals of military service and homecoming experiences have been done on World War II veterans. However, Spiro et al. (2016) hypothesized that there were war cohort differences in military experiences that could affect life-long adaptation. For example, Boscarino et al. (2018) found that Vietnam veterans reported less welcoming homecoming experiences than OEF/OIF/OND veterans. We examined war cohort differences among OEF/OIF/OND, Persian Gulf, and Vietnam combat veterans in military service appraisals and homecoming experiences. We used pilot data from Veterans Aging: Longitudinal studies in Oregon (VALOR) from an online survey. The sample included male and female combat veterans (Mage = 58.1, SD = 12.0, range = 35-83, 30.5% female): 39 from the OEF/OIF/OND, 68 from the Persian Gulf War, and 60 from the Vietnam War cohorts. Comparable to earlier studies (e.g., Aldwin et al., 1994), combat veterans were surprisingly much more likely to endorse desirable appraisals than the undesirable ones, with each of the 14 desirable appraisals endorsed by over 90% of the veterans. Fewer endorsed the undesirable experience items; the most common was separation from loved ones and loss of friends. Most also reported positive homecoming experiences. Contrary to expectations, ANOVAs revealed that there were no significant differences in appraisals of desirable and undesirable military service experiences, nor in homecoming experiences among the war cohorts. In this small sample, military experiences were perceived similarly among combat veterans despite differences in wartime experiences. Most felt that positive experiences resulted from their desirable military service.

